# Cross Sectional E-Health Evaluation Study for Telemedicine and M-Health Approaches in Monitoring COVID-19 Patients with Chronic Obstructive Pulmonary Disease (COPD)

**DOI:** 10.3390/ijerph18168513

**Published:** 2021-08-12

**Authors:** Abdullah H. Alsharif

**Affiliations:** Department of Management Information Systems, College of Business Administration-Yanbu, Taibah University, Yanbu, Saudi Arabia; alsharifa@taibahu.edu.sa

**Keywords:** mHealth, Telehealth, COPD, usefulness, evaluation, satisfaction

## Abstract

Monitoring COVID-19 patients with COPD has become one of the major tasks in preventing transmission and delivering emergency healthcare services after vaccination in case of any issues. Most COVID-19-affected patients are suggested to self-quarantine at home or in institutionalized quarantine centers. In such cases, it is essential to provide remote healthcare services. For remote healthcare monitoring, two approaches are being considered in this study, which include mHealth and Telehealth. A mixed-methods approach is adopted, where survey questionnaires are used for collecting information from 108 patients and semi-structured interviews are used with seven physicians regarding mHealth and Telehealth approaches. Survey results indicated that mHealth is rated to be slightly more effective than Telehealth, and interview results indicated that Telehealth is identified to be slightly more effective than mHealth in relation to parameters including usefulness, ease of use and learnability, interface and interaction quality, reliability, and satisfaction. However, both physicians and patients opined that both mHealth and Telehealth have a promising future with increasing adoption. Based on the findings, it can be concluded that both mHealth and Telehealth are considered to be effective in delivering remote care for COPD patients infected with COVID-19 at home. Implications of the study findings are discussed.

## 1. Introduction

COVID-19, since its identification in December 2019, has been affecting many people across the world, resulting in different waves of rising infections and deaths. As of 9 May 2021, there are 1.5 billion confirmed COVID-19 cases, including 3 million deaths reported globally [1]. The vast majority COVID-19 cases are mainly found in the West, including the Americas (63 million), followed by Europe (52 million). However, a steep rise in the number of cases was identified in the past 2 months in Southeast Asia (25 million), especially India (22 million) [1]. The number of deaths related to COVID-19 has been on the surge, with more than 570,000 reported deaths in the USA [2], 419,114 deaths in Brazil [3], and 242,362 deaths in India [4]. The rise in the number of COVID-19-related deaths were attributed to older age of patients and previous health complications such as diabetes, blood pressure, asthma and other critical health conditions which increase the risk of death among patients [5,6,7,8,9]. One such health condition wherein COVID-19 can have a serious impact on people is chronic obstructive pulmonary disease (COPD). It is identified that COVID-19 patients with COPD have a high risk of admission to an intensive care unit, mechanical ventilation, or death [10]. In addition, levels of angiotensin-converting enzyme 2 (ACE2), the reported host receptor of the virus responsible for COVID-19 (SARS-CoV-2), have been observed to be increased in patients with COPD [11]. Given the severity associated with COVID-19-infected COPD patients, there is increasing pressure on the health system to provide additional care. There is a need to maintain and reinforce follow-up and close management for these patients, with the aim of limiting collateral effects that could be induced by non-optimal management of COPD during and after the pandemic [12]. Strategies such as increased vaccination can help in preventing the mortality rate of COVID-19 infected COPD patients. However, in spite of administering over 11 billion vaccine doses globally [1], there is still a considerable global population that needs access to vaccines, especially in low- and middle-income group countries [13,14]. However, despite vaccination and other implementation of preventive and mitigation strategies such as lockdowns, increasing vaccination, etc. the new cases continue to rise globally. The risk of drug shortage due to COVID-19 restrictions is another major factor that can affect the treatment of COVID-19-infected patients with COPD and other critical respiratory conditions such as asthma, diabetes, etc. [15]. Moreover, the complications after vaccination were identified to be mild to moderate, which include pain, fever, headache, diarrhea, etc. [16,17]. Recently, black fungal infection (mucormycosis) is on the rise in Southeast Asia, resulting in a rising number of deaths and blindness among the vaccinated [18,19]. Therefore, there is an increasing need to monitor COVID-19 patients after treatment and vaccinated people, which can significantly increase the burden on healthcare systems.

Considering these factors, the best available options for managing the increased burden of managing COVID-19 patients in home quarantine and vaccinated people with additional health conditions such as COPD is to adopt effective remote monitoring of the patients’ conditions and adopt effective health information management techniques [20]. Effective information management techniques including real-time monitoring, data storing, transfer, retrieval, and update, are essential for improved clinical decision-making. Timely response to chronic conditions can help in preventing mortalities and provide quality care to the patients [21,22]. It has been identified that medically necessary, time-sensitive procedures can efficiently manage resources and improve clinical decision-making for treating COVID-19 patients [23]. Advances in technology have helped in developing various monitoring systems such as smart watches and diabetes monitors that can be integrated with mobile applications and transfer real-time data to hospital servers, improving the effectiveness of remote monitoring by efficiently managing the health information of the patients. eHealth, for instance, is an emerging field at the intersection of medical informatics, public health, and business, referring to the health services and information delivered or enhanced through the internet and related technologies [24]. mHealth and Telehealth are two major eHealth approaches that are being extensively used during the COVID-19 pandemic to deliver remote healthcare services. mHealth is a medical and public health practice supported by mobile devices, such as mobile phones, patient monitoring devices, personal digital assistants (PDAs), and other wireless devices [25], whereas telemedicine is the use of medical information exchanged from one site to another via electronic communications to improve a patient’s clinical health status [26]. While Telehealth is mainly used for information exchange, mHealth can be used for both information exchange and remote monitoring and diagnosis. The difference between both approaches lies in the modes of information transmission. In mHealth, the information is transferred through mobile applications, while in Telehealth, information has to be communicated by the patients to the medical representative. 

In relation to chronic diseases, such as COPD, associated with COVID-19, parameters such as oxygen levels, electrocardiograms (ECGs), carbon dioxide partial pressure (PaCO2), etc. can be monitored remotely (mHealth) using sensors and devices linked to the patients mobile, which transmits information to hospitals through the mobile applications [27]. The cost of care assistance for chronic diseases such as COPD are dramatically increasing, as a result of which remote healthcare management systems such as Telehealth can be used to reduce costs and increase healthcare efficiency [28]. Therefore, remote monitoring solutions such as mHealth and Telehealth can be considered as an alternative to traditional healthcare operations, which not only decreases the burden on the already strained healthcare sector due to COVID-19, but also improves healthcare information management and delivers quality care for patients. In this context, it was observed that mHealth technologies could help in mitigating the effects of the COVID-19 pandemic [29] by increasing the reach of local guidance for healthcare professionals in managing COVID-19 outbreak and treatment procedures [30], and in coordinating mHealth infrastructure for managing the COVID-19 pandemic [31]. Studies [32,33,34,35] have evaluated mHealth applications in relation to various parameters, including usefulness, ease of use, learnability, interface and interaction quality, reliability, satisfaction, and future use, etc., which reflected positive outcomes. Similarly, Telehealth approaches were also identified to be effective in relation to the above-mentioned parameters [36]. 

Both mHealth and Telehealth approaches have some advantages and drawbacks. For instance, patients at home may not regularly monitor their health condition and may not enter the health data into the mobile applications, which may lead to ineffective remote monitoring. However, the Telehealth approach may address this issue, as healthcare practitioners may call the patients and regularly collect the data and provide instant feedback. However, repeated calls from the healthcare professionals may create discomfort for the patients. However, for effective healthcare management, the need for accurate and daily health data is essential, and both mHealth and Telehealth can serve this purpose. However, there is a lack of research on comparing both approaches in remote monitoring of COPD patients infected with COVID-19 in home quarantine or vaccinated patients at home. Therefore, the purpose of this study is aimed to evaluate Telehealth and mHealth approaches in monitoring (remote monitoring of exercise tolerance, comorbidity, and smoking habits, oxygen levels, blood pressure, sugar levels, and other factors, as prescribed by the patients’ respective hospitals) COVID-19 patients with COPD after the treatment and vaccination.

## 2. Materials and Methods

The purpose of this study is to compare and evaluate the mHealth and Telehealth approaches in monitoring COVID-19 patients with COPD after treatment and vaccination while at home. A mixed-methods approach employing both qualitative (semi-structured interviews) and quantitative (questionnaire-based survey) were adopted for collecting the data regarding mHealth and Telehealth approaches from physicians and patients, respectively, in Saudi Arabia. 

### 2.1. Questionnaire Design

As discussed in the introduction section, there are various parameters used for evaluating both mHealth and Telehealth applications [32,33,34,35,36]. To cover different contexts of evaluation, Telehealth Usability Questionnaire (TUQ) is adopted in this study, including items related to various parameters (usefulness, ease of use, interface quality, interaction quality, reliability, satisfaction, and future use) from [36]. TUQ is preferred in this study because it covers wide range of parameters that are used individually in various studies [29,30,31,32], making a comprehensive list of items to be used in evaluating Telehealth applications. Furthermore, items from (MAUQ) [37], including ease of use, satisfaction, usefulness, are considered along with TUQ for developing questionnaires for mHealth and Telehealth evaluation, respectively, as shown in Appendix A. Both questionnaires (mHealth and Telehealth) have same set of questions with parameters including usefulness (three items), ease of use and learnability (three items), interface quality (three items), interaction quality (three items), reliability (three items), and satisfaction and future use (four items). Multiple-choice answers and five-point Likert scale ratings [38] were used for answering the questions by the participants. The questionnaire was initially designed in English (a copy of survey questionnaire is presented in Appendix A), which was then translated to Arabic using two professional Arabic translators. The Arabic version questionnaire was designed using QuestionPro application, and a survey link was generated for accessing the survey. A pilot study was conducted with 12 randomly selected patients from Saudi Arabia for evaluating the questionnaire. Based on the feedback from pilot study participants, few changes were made in relation to the questions’ formulation and grammatical errors in Arabic. In addition, Cronbach’s alpha for all items in in the questionnaire was identified to be greater than 0.81, revealing good consistency and reliability. In addition, the interview questionnaire included eight questions reflecting the interviewees’ experiences with Telehealth and mHealth, ease of use, satisfaction and future use, usefulness, learnability, interface quality, interaction quality, and reliability (as shown in Appendix B). Thus, semi-structured interviews were adopted in order to evaluate both mHealth and Telehealth from the perspectives of healthcare practitioners, and a survey instrument was adopted to evaluate both approaches from perspectives of patients, reflecting the two main actors in remote monitoring in healthcare system. 

### 2.2. Recruitment

COPD patients diagnosed with COVID-19 and vaccinated COPD patients were recruited for the survey using the survey link generated using QuestionPro application. The survey link was forwarded to the patients through emails and other social media platforms such as Facebook and WhatsApp. The survey was conducted for a period of 5 weeks from 15 April to 20 May 2021. Physicians were contacted through emails and over phone, requesting them to participate in the interviews. Interviews were scheduled from 16 April to 5 May 2021, and were conducted online. On average, each interview lasted for 35 min. 

### 2.3. Sampling

Considering the purpose and objective of the study, which is to collect the data from a specific group of population (COPD patients diagnosed with COVID-19 and vaccinated COPD patients at home involved in remote monitoring), a purposive sampling approach was adopted [39]. Accordingly, a nonprobability sample was obtained based on the objective of the study, which mainly focused on analyzing the patients’ and physicians’ perceptions of mHealth and Telehealth. The survey link was initially forwarded 142 patients using various online channels. A total of 108 students participated in the survey, reflecting a response rate of 76.05%. While 19 physicians were contacted for semi-structured interviews, 7 accepted invitations and took part in the interviewees. Low response rate in interviews is due to the busy schedules of the physicians owing to the COVID-19 outbreak.

### 2.4. Data Analysis

The survey was developed using Google forms and conducted for a period of 5 weeks. Both survey and interview data are analyzed and discussed using nine themes, which included experiences, usefulness, ease of use, learnability, interface quality, interaction quality, reliability, satisfaction, and future use. In relation to survey data, relative frequencies for each item under these themes and statistical tests (*t*-tests) are used for analyzing the data, which are presented in the following section.

## 3. Results and Discussion

### 3.1. Survey Results

The final sample achieved for the study was 108. The demographic information of the participants is presented in Table 1. Among the total participants, 58.3% were male (63/108), and 41.7% were female (45/108). Considering the age groups, 43.5% were aged between 35 and 44 years (47/108), followed by 30.6% between 25 and 34 years (114/479), 12% between 18 and 24 years (13/108), 12% between 45 and 54 years (13/108), and 1.9% participants aged 55 or more than 55 years (2/108). Focusing on the education of the participants, 38.9% were bachelor’s degree graduates (42/108), followed by 30.6% master’s graduates (33/108), 15.7% high school graduates or diploma graduates (17/108), and 14.8% doctorates (16/108). Demographics of the participants reflected good participation levels by both genders. Moreover, the majority of the participants were aged between 25 and 44 years, reflecting the population who is better equipped with the skills of using health information technologies [40,41,42], and has good education levels.

All the participants were having an experience of using both Telehealth and mHealth approaches. In relation to the experience of using Telehealth (via telephone/mobile), 65.7% of the participants had 2 or less than 2 years of experience (71/108), followed by 17.6% having 2 to 5 years of experience (19/108), 9.3% having 10 or more years of experience (10/108), and 7.4% having 5 to 10 years of experience (8/108). In relation to the experience of using mHealth (via mobile application/smart sensors), 63.9% of the participants had 2 or less than 2 years of experience (69/108), followed by 25.9% having 2 to 5 years of experience (28/108), 6.5% having 10 or more years of experience (7/108), and 3.7% having 5 to 10 years of experience (4/108). The experience levels of the participants in relation to mHealth and Telehealth reflected almost similar statistics, with the majority of them having 2 years or less and 2 to 5 years of experience.

Participants’ opinions on the usefulness of both mHealth and Telehealth are presented in Table 2. Improved access to healthcare was identified to be a highly rated factor related to usefulness, followed by the time-saving factor, and approaches meeting the healthcare needs of the users. Both approaches were identified to be similarly rated by the participants in relation to the usefulness parameter.

Furthermore, to identify the differences of opinions in relation to mHealth and Telehealth, a *t*-test was conducted, as shown in Table 3. The mean scores of mHealth (Mean = 3.64, SD = 1.12) and Telehealth (Mean = 3.61, SD = 1.08), identified in the analysis, reflected that participants found that both mHealth and Telehealth approaches to be effective in terms of usefulness. *t*-value, as shown in Table 3, was found to be (*t* = 0.2004) at 0.05 confidence interval, and was identified as not statistically significant (*p* > 0.05). Therefore, no significant differences of opinions in relation to the usefulness of mHealth and Telehealth can be observed. The findings are similar to [32], reflecting the usefulness of both approaches. As both approaches are aimed at improving access to healthcare and save time in accessing healthcare needs, it is possible that both approaches are identified to be useful by the participants.

Participants’ opinions on the ease of use and learnability parameters are presented in Table 4. It can be observed that simple to use and easy to learn factors of mHealth are slightly greater than that of Telehealth; no major differences were identified in relation to the ability of the approach for enhancing productivity. While the mHealth application is mobile-based, and easy to use, Telehealth completely relies on calls in providing care and monitoring health information. Therefore, slight differences in terms of ease of use and learnability factors can be expected.

Furthermore, to identify the differences of opinions in relation to mHealth and Telehealth, a *t*-test was conducted, as shown in Table 5. The mean scores of mHealth (Mean = 3.72, SD = 1.12) and Telehealth (Mean = 3.69, SD = 1.1), identified in the analysis, reflected that participants found that both mHealth and Telehealth approaches to be effective in terms of ease of use and learnability. *t*-value, as shown in Table 5, was found to be (*t* = 0.1986) at 0.05 confidence interval, and was identified as not statistically significant (*p* > 0.05). Therefore, no significant differences of opinions in relation to ease of use and learnability parameters of mHealth and Telehealth can be observed. These findings can be related to [33], in which ease of use and learnability were rated to be effective by the majority of the participants. 

While mHealth applications have a mobile interface, Telehealth does not have any physical interface, but the quality of the interface can be identified from the communication between patients and healthcare practitioners over telephone or mobiles. In relation to the interface quality, both mHealth and Telehealth approaches were rated slightly above average (Mean = 2.5), as shown in Table 6. It is interesting to note that only 50.9% of the participants either strongly agreed or agreed that the Telehealth approach meets their healthcare needs, and only 52.7% of the participants either strongly agreed or agreed that the mHealth approach meets their healthcare needs, reflecting that there is a considerable number of participants who are not happy with interface quality in both approaches.

Furthermore, to identify the differences of opinions in relation to mHealth and Telehealth, a *t*-test was conducted, as shown in Table 7. The mean scores of mHealth (Mean = 3.42, SD = 1.05) and Telehealth (Mean = 3.43, SD = 1.06), identified in the analysis, reflected that participants found that both mHealth and Telehealth approaches to be effective in terms of interface quality. *t*-value, as shown in Table 7, was found to be (*t* = 0.0697) at 0.05 confidence interval, and was identified as not statistically significant (*p* > 0.05). Therefore, no significant differences of opinions in relation to interface quality parameters of mHealth and Telehealth can be observed. These findings related to mHealth are similar to [34], indicating good interface quality of mHealth applications.

In relation to interaction quality, no significant differences were identified between mHealth and Telehealth, as identified from Table 8. In relation to the ability of the approaches reflecting in similar to personal interactions, less than 50% of the participants reflected the opinion that these approaches are similar to personal interactions. Moreover, more than 50% of the participants stated that they are not able to express their opinions effectively on mHealth and Telehealth applications. 

Furthermore, to identify the differences of opinions in relation to mHealth and Telehealth, a *t*-test was conducted, as shown in Table 9. The mean scores of mHealth (Mean = 3.41, SD = 1.05) and Telehealth (Mean = 3.40, SD = 0.99), identified in the analysis, reflected that participants found that both mHealth and Telehealth approaches to be effective in terms of interaction quality. *t*-value, as shown in Table 9, was found to be (*t* = 0.0720) at 0.05 confidence interval, and was identified as not statistically significant (*p* > 0.05). Therefore, no significant differences of opinions in relation to interface quality parameters of mHealth and Telehealth can be observed. These findings related to mHealth are similar to [33,34], indicating average interaction quality of mHealth and Telehealth approaches.

In relation to reliability (Table 10), the mHealth approach was rated as slightly better than the Telehealth approach, stating that the approach was similar to hospital visits in delivering the care, and also in the ability to fix issues by receiving messages through the application (in comparison to messages received through calls). The majority of the participants (>50%) were identified to be neutral (neither agree nor disagree) in relation to all the factors listed in the reliability parameter. 

Furthermore, to identify the differences of opinions in relation to mHealth and Telehealth, a *t*-test was conducted, as shown in Table 11. The mean scores of mHealth (Mean = 3.27, SD = 1.03) and Telehealth (Mean = 3.08, SD = 1.05), identified in the analysis, reflected that participants found that both mHealth and Telehealth approaches to be effective in terms of reliability. *t*-value, as shown in Table 11, was found to be (*t* = 1.3424) at 0.05 confidence interval, and was identified as not statistically significant (*p* > 0.05). Therefore, no significant differences of opinions in relation to the reliability parameter of mHealth and Telehealth can be observed. Considering the less acceptance of eHealth in Saudi Arabia, due to various factors of influence, it may be possible that low responses were identified in relation to reliability factor, which can be compared to the findings in [43]. 

In relation to satisfaction (Table 12), it can be identified that participants were slightly more satisfied with mHealth compared to Telehealth across all the factors related to satisfaction and future use. Considering the overall satisfaction, there is no difference of opinions expressed in relation to both approaches. However, while 60% of the participants agreed that they would use mHealth in the future, 64% of the participants stated they would use Telehealth in the future, indicating a slightly more preference towards Telehealth over mHealth.

Furthermore, to identify the differences of opinions in relation to mHealth and Telehealth, a *t*-test was conducted, as shown in Table 11. The mean scores of mHealth (Mean = 3.63, SD = 1.06) and Telehealth (Mean = 3.53, SD = 1.04), identified in the analysis, reflected that participants found that both mHealth and Telehealth approaches to be effective in terms of satisfaction and future use. *t*-value, as shown in Table 13, was found to be (*t* = 0.6998) at 0.05 confidence interval, and was identified as not statistically significant (*p* > 0.05). Therefore, no significant differences of opinions in relation to the reliability parameter of mHealth and Telehealth can be observed.

These findings may be compared to [31,32,40] in relation to satisfaction levels. Moreover, preference over these approaches may be influenced by the recent COVID-19 outbreak, which has led to increased adoption of eHealth approaches due to the surge in COVID-19 cases and preventive measures such as lockdowns and curfews. However, lack of reliability as identified in [43] can be one of the reasons for leaning more towards Telehealth rather than mHealth.

### 3.2. Interview Results

A total of seven healthcare practitioners were interviewed, and all of them were males. Among them, three participants belonged to the age group of 35–44 years; another three in 45–54 years; and one participant in 25–34 years. Four participants were general physicians, one participant was a dentist, one was a surgery specialist, and another was a medical specialist. Three participants had experience of 2 or less years in using mHealth and Telehealth approaches, two had an experience of 2 to 5 years, and another two had an experience of 5 to 10 years. The participants’ experience levels and roles reflect a good sample for collecting the information about mHealth and Telehealth.

Focusing on the opinions expressed in relation to Telehealth, all the participants identified good usefulness levels for Telehealth. One of the interviewees identified it to be an easier approach to reach patients without any difficulty or requirement to learn new technologies, reflecting the edge over mHealth. Another interviewee identified Telehealth to be an effective approach in providing distant care. In relation to mHealth, only one participant identified its usefulness to be poor, while the rest of them indicated good levels of usefulness. One of the interviewees identified it to be very useful, providing real-time information at any time. The findings in relation to usefulness indicated that the majority of the participants identified both approaches to be of good usefulness. However, Telehealth was identified to be slightly more useful compared to mHealth.

In relation to ease of use, all the interviewees mentioned Telehealth to be very easy to use and learn. However, one of the interviewees mentioned that additional training relating to compliances and standards is required in using Telehealth. One of the interviewees mentioned that Telehealth may not be easy to learn in the beginning, but as handling of it improves, one can learn effectively. Similarly, all the interviewees identified mHealth to be easy to use, with little knowledge of computers and technology. However, one of the interviewees mentioned that experience is required in using mHealth, and another interviewee mentioned that mHealth is hard to use. Another interviewee mentioned that mHealth may be effective for learning for only those who use mobiles more frequently, and have experience of using applications. The overall analysis of responses indicated that Telehealth was rated slightly more than mHealth in terms of ease of use and learnability.

In relation to interface and interaction quality, all the interviewees reflected Telehealth to be good. Similarly, in relation to mHealth, only one interviewee identified it with poor interface and interaction quality. Using Telehealth was identified to be effective because interaction using Telehealth takes comparatively less time than visits, which can save time; while using mHealth, it was indicated that a lot of time could be saved as it is one-way messaging. Both approaches indicated time-saving as an outcome of effective interface and interaction quality. However, Telehealth was slightly rated more than mHealth.

Focusing on the reliability parameter, all the interviewees indicated Telehealth to be reliable but extended their statement that reliability may depend on many factors, including the physician and patient and the technology used to connect them. While Telehealth mainly relies on calls, it is sometimes possible that internet technologies such as voice over internet protocol or applications such as skype or zoom may be used, which may raise concerns over security and privacy. However, in relation to mHealth, five interviewees stated it to be reliable. One of the interviewees stated that more research is needed to assess its reliability, while others raised privacy and security concerns over mHealth.

Findings relating to usefulness, ease of use, learnability, interface and interaction quality, and reliability from interviewees’ perspectives reflected a slightly greater preference towards Telehealth compared to mHealth in contrast to patients’ perspectives. The differences of opinions among the participants may be related to their experience and understanding of these approaches, and also the features and design of the applications they have been using, which can influence their perspectives [44,45,46].

In relation to the satisfaction parameter, all the interviewees reflected good satisfaction levels about the Telehealth approach. However, focusing on the mHealth approach, one interviewee stated moderate satisfaction and another interviewee stated poor satisfaction. Findings reflected that the interviewees are slightly more satisfied with Telehealth compared to mHealth. These results regarding satisfaction contrasted with survey results, where participants identified with being slightly more satisfied with mHealth compared to Telehealth, supporting findings from [47,48,49,50]. Furthermore, in relation to future use prospects, Telehealth was identified to be a promising approach in reducing clinical visits, improving quality healthcare. Similarly, mHealth was also identified to be having a promising future where electronic health records can be integrated with daily monitoring systems, providing 24 × 7 remote healthcare services which can significantly improve effectiveness and efficiency of care. The results have indicated that both mHealth and Telehealth would be increasingly adopted in the future, similar to survey results.

## 4. Conclusions

This study has compared and evaluated mHealth approaches in remote monitoring of COPD patients diagnosed with COVID-19. The importance of this study arises from the rising complications and effects of COVID-19 after the recovery and during the home quarantine, associated with the additional complications of COPD condition. To evaluate the approaches, both patients’ and physicians’ perspectives are considered. The findings have indicated that patients’ views were in contrast to physicians’ views. While patients leaned towards mHealth, physicians leaned towards Telehealth. However, both approaches were identified to be effective in terms of their usefulness, ease of use and learnability, interface and interaction quality, reliability, satisfaction, and future use, with minor differences. This study has few limitations. As different patients use different mHealth applications and adopt different practices in Telehealth, there could be a certain bias in the results. In addition, the number of participants in both survey and interviews was lower due to the impact of the COVID-19 outbreak. Therefore, generalizations should be made with care. This study has both theoretical and practical implications. First, this study addresses the gaps in the literature in evaluating the remote monitoring approaches in the context of COVID-19 pandemic. Second, the findings can be used to improve the mHealth and Telehealth approaches in relation to the needs of the patients and physicians. Moreover, as the study has been conducted in Saudi Arabia, the findings can only be compared to the population with similar demographics. Therefore, future research may focus on the evaluation of different remote monitoring approaches in different regions reflecting varying demographics.

## Figures and Tables

**Table 1:** Frequency distribution of demographic variables.

Variables	*n* (%)
**Gender**	
Male	63 (58.3%)
Female	45 (41.7%)
**Age**	
18–24	13 (12%)
25–34	33 (30.6%)
35–44	47 (43.5%)
45–54	13 (12%)
55 and above	2 (1.9%)
**Education**	
High school graduate, diploma, or equivalent	17 (15.7%)
Bachelor’s degree	42 (38.9%)
Master’s degree	33 (30.6%)
Doctorate	16 (14.8%)

**Table 2:** Usefulness of mHealth and Telehealth.

Items	mHealth		Telehealth	
	Mean	Std.Dev	Mean	Std.Dev
Access to healthcare services	3.75	1.10	3.67	1.08
Saves time	3.68	1.17	3.65	1.13
Meets healthcare needs	3.49	1.09	3.53	1.03

**Table 3:** Difference in usefulness of mHealth and Telehealth.

Variable	Approach	*n*	Mean	Std.Dev	*df*	*t*-Value	*p*-Value
Usefulness	mHealth	108	3.64	1.12	214	*0.2004*	*0.8414*
	Telehealth	108	3.61	1.08			

**Table 4:** Ease of use and learnability of mHealth and Telehealth.

Items	mHealth		Telehealth	
	Mean	Std.Dev	Mean	Std.Dev
Simple to use	3.70	1.09	3.62	1.15
Easy to learn	3.75	1.04	3.73	1.11
Productivity	3.72	1.13	3.73	1.04

**Table 5:** Difference in ease of use and learnability of mHealth and Telehealth.

Variable	Approach	*n*	Mean	Std.Dev	*df*	*t*-Value	*p*-Value
Ease of use and Learnability	mHealth	108	3.72	1.12	214	*0.1986*	*0.8428*
	Telehealth	108	3.69	1.1			

**Table 6:** Interface quality of mHealth and Telehealth.

Items	mHealth		Telehealth	
	Mean	Std.Dev	Mean	Std.Dev
Pleasant	3.5	1.04	3.48	1.07
Simple and easy to understand	3.55	1.03	3.61	1.06
Ability to do according to users’ needs	3.22	1.10	3.20	1.05

**Table 7:** Difference in interface quality of mHealth and Telehealth.

Variable	Approach	*n*	Mean	Std.Dev	*df*	*t*-Value	*p*-Value
Usefulness	mHealth	108	3.42	1.05	214	*0.0697*	*0.9445*
	Telehealth	108	3.43	1.06			

**Table 8:** Interaction quality of mHealth and Telehealth.

Items	mHealth		Telehealth	
	Mean	Std.Dev	Mean	Std.Dev
Easy to talk to clinician	3.46	1.01	3.48	0.98
Able to express effectively	3.5	1.02	3.50	1.07
Similar to personal interaction	3.28	1.13	3.23	1.01

**Table 9:** Difference in interaction quality of mHealth and Telehealth.

Variable	Approach	*n*	Mean	Std.Dev	*df*	*t*-Value	*p*-Value
Usefulness	mHealth	108	3.41	1.05	214	*0.0720*	*0.9427*
	Telehealth	108	3.40	0.99			

**Table 10:** Reliability of mHealth and Telehealth.

Items	mHealth		Telehealth	
	Mean	Std.Dev	Mean	Std.Dev
Approach was similar to in-person visits	3.16	1.03	2.96	1.09
Ability to recover from the mistakes in the system	3.37	1.03	3.28	1.03
Ability of the system in sending messages to fix issues	3.28	1.04	3	1.02

**Table 11:** Difference in Reliability of mHealth and Telehealth.

Variable	Approach	*n*	Mean	Std.Dev	*df*	*t*-Value	*p*-Value
Usefulness	mHealth	108	3.27	1.03	214	*1.3424*	*0.1809*
	Telehealth	108	3.08	1.05			

**Table 12:** Satisfaction and future use of mHealth and Telehealth.

Items	mHealth		Telehealth	
	Mean	Std.Dev	Mean	Std.Dev
Comfortability	3.50	1.08	3.46	1.04
Acceptable way to receive healthcare services	3.60	1.06	3.55	1.03
I would use in future	3.71	1.06	3.57	1.05
Overall satisfaction	3.71	1.05	3.57	1.05

**Table 13:** Difference in satisfaction and future use of mHealth and Telehealth.

Variable	Approach	*n*	Mean	Std.Dev	*df*	*t*-Value	*p*-Value
Usefulness	mHealth	108	3.63	1.06	214	*0.6998*	*0.4848*
	Telehealth	108	3.53	1.04			

## Data Availability

Not applicable.

## Appendix A

**Evaluating Telehealth and mHealth approaches in monitoring the COVID-19 patients with chronic obstructive pulmonary disease (COPD): Survey Questionnaire (Telehealth)**

1. Name.
2. Gender: Male/Female.
3. Education: Associate’s degree, Bachelor’s degree, Completed some postgraduate, Master’s degree, Ph. D.
4. Experience with Telehealth: None, Less than 3 months, 3–6 months, 6 months–1 year, more than 1 year.
5. Please rate the following aspects of the health system on a scale of one to five (1: Strongly Disagree; 2: Disagree; 3: Neutral; 4: Agree; 5: Strongly Agree).

## Appendix B

**Evaluating Telehealth and mHealth approaches in monitoring COVID-19 patients with chronic obstructive pulmonary disease (COPD): Survey Questionnaire (mHealth)**

1. Name.
2. Gender: Male/Female.
3. Education: Associate’s degree, Bachelor’s degree, Completed some postgraduate, Master’s degree, Ph. D.
4. Experience with Telehealth: None, Less than 3 months, 3–6 months, 6 months–1 year, more than 1 year.
5. Please rate the following aspects of the health system on a scale of one to five (1: Strongly Disagree; 2: Disagree; 3: Neutral; 4: Agree; 5: Strongly Agree).

## Appendix C

**Interview Questionnaire**

**Telehealth**

1. Please reflect your opinions on the usefulness of Telehealth.
2. Please reflect your opinions on the ease of use of Telehealth.
3. Please reflect your opinions on the learnability of Telehealth.
4. Please reflect your opinions on the interface quality of Telehealth.
5. Please reflect your opinions on the interaction quality of Telehealth.
6. Please reflect your opinions on the reliability of Telehealth.
7. Please reflect your opinions on the overall satisfaction of Telehealth.
8. Please reflect your opinions on the future use of Telehealth.

**mHealth**

1. Please reflect your opinions on the usefulness of mHealth.
2. Please reflect your opinions on the ease of use of mHealth.
3. Please reflect your opinions on the learnability of mHealth.
4. Please reflect your opinions on the interface quality of mHealth.
5. Please reflect your opinions on the interaction quality of mHealth.
6. Please reflect your opinions on the reliability of mHealth.
7. Please reflect your opinions on the overall satisfaction of mHealth.
8. Please reflect your opinions on the future use of mHealth.
